# Novel Dimethylacetamide-Containing Formulation Improves Infraorbital Anaesthesia Efficacy in Rats with Periodontitis

**DOI:** 10.1155/2020/3058735

**Published:** 2020-04-14

**Authors:** Ekaterina V. Blinova, Evgeniia V. Shikh, Elena V. Semeleva, Aleksandra M. Yurochkina, Andrey V. Novikov, Anna P. Vediaeva, Arkadii B. Lebedev, Elena G. Lobanova, Olga V. Vasilkina, Dmitry S. Blinov, Yan A. Mazov, Evgeniia A. Kogan

**Affiliations:** ^1^Department of Operative Surgery and Clinical Anatomy, Department of Pathology, Department of Dentistry, Sechenov University, Moscow 119991, Russia; ^2^Department of Dentistry, Department of Ophthalmology, National Research Mordovia State University, Saransk 430005, Russia; ^3^Department of Pharmaceutical Chemistry and Drug Design, All-Union Scientific Center of Biological Active Compounds, Staraja Kupavna 142450, Russia; ^4^Department of Pharmacology, Tver State Medical University, Tver 170100, Russia; ^5^Department of Pharmacology, A. I. Yevdokimov Moscow State University of Medicine and Dentistry, Moscow 119983, Russia

## Abstract

**Background:**

To evaluate acute toxicity and local anaesthetic activity of a formulation containing a novel dimethylacetamide derivative, antioxidant, and vasoconstrictor in rats with chronic periodontitis.

**Methods:**

Novel anaesthetic dimethylacetamide-containing formulation LHT-15-32 was studied as 2% water solution. Its acute intravenous and subcutaneous toxicity was determined in mice. Pain sensitivity threshold of the upper second molar was determined in rats with experimental periodontitis. Oxidative stress activity and total antioxidant capacity were determined in rats' gingival mucosa by induced chemiluminescence. Local changes were evaluated in periodontal tissue by morphological examination. Tissue IL-1*β*, IL-10, and TNF-α concentration was quantitatively assessed by an enzyme-linked immunosorbent assay. LHT-15-31 Na-blocking activity was studied on isolated neurons of *Limnaea stagnalis*' parapharyngeal ganglion. Isolated sciatic nerve of *Rana radibunda* was perfused with different concentrations of LHT-15-32 to assess its conductivity. Statistical analysis was used, and continuous variables were presented as mean ± square deviation. The normality of distribution was determined using ANOVA. Newman–Keuls parametric criterion was used for intergroup comparison. LD_50_ indexes were calculated by probit analysis.

**Results:**

LHT-15-32 acute intravenous and subcutaneous toxicity was lower than that of its active substance. The formulation by infraorbital administration induced deep dental anaesthesia which lasted over 70 min and activated the local antioxidant defense system and decreased IL-1*β* level in gingival tissue. LHT-15-32 triggered tissue reparation around the impacted upper molar in rats assessed five days after administration. At 10^−6^ to 10^−3^ M concentration, LHT-15-32 inhibited sciatic nerve conductivity and blocked Na^+^ channels of isolated neurons in a dose-dependent manner.

**Conclusions:**

The formulation may be considered as an effective and safe approach to anaesthetize upper molars with periodontitis.

## 1. Introduction

Modern clinical practice, despite obvious success in creating new local anaesthetics (LA) as well as optimizing technologies for delivering drugs to the site of exposure, continues to experience difficulties in achieving optimal analgesic effect of LA [1]. One of the most frequent reasons for the lack of LA efficacy in dentistry is that almost always LA are administered to patients with inflammatory processes in the area of tooth pulp, periapical zone, periodontal, or alveolar region [1, 2].

Practitioners often experience great difficulties with the induction of sufficient depth and duration of tooth anaesthesia with inflammation when using the infiltration method or nerve blockade during pulpitis or apical periodontitis [3]. Clinical studies demonstrate failure in 30–45% or low success in 19–56% of patients with inflammatory process of the lower molars during inferior alveolar nerve blockade even in case of anaesthesiological benefits produced by an experienced clinician [4]. Neither the combination of infiltration anaesthesia of cheek and tongue, nor the traditional block of the nerve is accompanied by an increase in the efficiency of manipulation. Thus, the problem of adequate anaesthesia in dentistry now is an important challenge for both clinical and experimental pharmacologists [1, 4].

Lidocaine has a long history of successful use as an LA since it has been first administered in the middle of the 20^th^ century [5]. Lidocaine inhibits nerve conductivity by reversible blockade of Na^+^ channels and stabilises the cellular membrane due to its antioxidant property. As a monotherapy or combining with vasoconstrictors, it has been showing satisfactory anaesthesia except in case of local inflammation [1].

The study's aim is to evaluate acute toxicity and LA activity of a formulation containing a novel dimethylacetamide derivative, antioxidant, and vasoconstrictor in rats with chronic periodontitis.

## 2. Materials and Methods

### 2.1. Ethics

The study has met all the requirements of GLP standards and the European Convention for the Protection of Vertebrates Animals used for Experimental and Other Scientific Purposes regulations. The study protocols have been reviewed and approved by the Sechenov University BioEthics Committee (Moscow, Russia) July 6, 2017 (meeting No. 7, reg. no. 6/7/2017-11).

### 2.2. Drug Formulation

Novel formulation (laboratory code: LHT-15-32) has been developed at the Department of Pharmaceutical Chemistry and Drug Design of All-Union Scientific Centre of Biological Active Compounds (AUSC BAC, Russia) as 2% water solution containing N-acetyl-L-glutamine 2-diethylamino-2′,6′-dimethylphenylacetamide (laboratory code: LHT-4-00, AUSC BAC; chemical purity: 99.75%) as an active substance. Every 1 mL of the solution contained 2 mg of ethyl-methyl-hydroxypyridine malate (EMHPM) and epinephrine 1 : 200,000. Pharmacological property of LHT-15-32 has been compared with LHT-4-00 alone solubilized in distilled water *ex tempore* and lidocaine (lidocaine HCI, 20 mg/mL, 20 mL, Hospira Inc., USA). Both the reference drugs have been studied at 2% concentration. Before introduction, pH of each test solution was determined and adjusted to 7.35 with 8.4% sodium bicarbonate considering both volume and concentration of the solutions.

### 2.3. Animals and Biological Objects

Fifty five white outbred mice weighing 18–20 g of both sexes and 15 frogs (*Rana radibunda*) were obtained from the Laboratory Animal Breeding Facility of Russian Academy of Sciences (RAS). Sixty five Sprague-Dawley male rats weighing 250–300 g were purchased at the Animals Breeding Facility of Institute of Theoretic and Experimental Biophysics of RAS (Russia). Animals were kept under pathogen-free condition and natural daylight cycles. Sterile food and water were provided *ad libitum*, and room temperature (25 ± 2°C) as well as humidity (60 ± 10%) were maintained. Mice were randomly divided into 11 equal groups for toxicology experiment. Fifty rats were randomly allocated into 5 groups of 10 animals in each: intact rats, control, lidocaine, LHT-4-00, and main group; fifteen animals were divided into 3 groups with 5 rats (intact, control, and main groups) and subjected to morphological examination. 3.5 cm length of frog's sciatic nerve specimens were isolated and kept in a Ringer solution for 30–40 min at 22°C before stimulation. Indifferent neurons were isolated from the parapharyngeal ganglion of *Limnaea stagnalis*.

### 2.4. Acute Toxicity Assay

LHT-15-32 acute intravenous (IV) toxicity was assessed as previously described by estimation of an average dose (LD_100_), which caused animals' death (Gad, 1990). The formulation subcutaneous (SC) toxicity (LD_50_) was registered in mice assigned to a definite dosage group 14 days after LHT-15-52 injection as previously described [6].

### 2.5. Modeling of Experimental Periodontitis

Experimental periodontitis was induced as described before in anaesthetized (thiopental sodium, Sandoz, GmbX, Austria; 40 mg/kg, IV) male Sprague-Dawley rats assigned to all the groups except one with intact animals by ligating the second upper molar with a silk thread (5-0, Ethicon Inc., USA) [7].

### 2.6. Dental Anaesthesia Assessment

On day 15, the anaesthetized animal (thiopental sodium, SANDOZ, GmbX, Austria; 40 mg/kg, IV) was placed on a heated platform of a stereotaxic system SR-5R (Narishige Co, Ltd, Japan) equipped with a portable drill. Two holes 0.3 mm in diameter and 3.0–3.5 mm in depth were drilled in the second upper molar, where free ends of a platinum-stimulating electrode (ADInstruments, USA) were fixed with a drop of plastic filling. The other ends of the electrode were drawn out through the hole in the cheek skin on the animal's withers and fixed with purse skin suture. Four days later, rectangular impulses (100 imp/s for 5 s) of increasing current strength from 0.1 to 10 mA (BIOPAC MP-160, BIOPAC Systems Inc., USA) were applied to the tooth to achieve a pain sensitivity threshold (PST) registered by occurrence of the animal motor reaction and vocalization. Test solution (0.2 mL) was administered infraorbitally 10 min after baseline PST registration (zero-level of PST). Full anaesthesia was suggested if 5 s-long 10 mA stimulation caused no animal reaction (100% level of PST).

### 2.7. Local Oxidative Stress Assay

We used Fe-induced chemiluminescence to assess oxidative stress activity (OSA) and total antioxidant capacity (TAC) in homogenates of gingival mucosa tissue of rats overdosed with thiopental sodium the next day after LA level measurement as previously described [8].

### 2.8. Enzyme-Linked Immunosorbent Assay (ELISA)

IL-1*β*, IL-10, and TNF-α concentrations were detected in rats' gingival tissue homogenates by quantitative ELISA using Rat Interleukin 1*β* (CSB-E08055r), Rat Interleukin 10 (CSB-04595r), and Rat TNF-α (CSB-11987r) ELISA Kits (Cusabio Technology, LLC, China) and a StatFax 4200 automatic reader (USA).

### 2.9. Sciatic Nerve Conductivity Assay

Sciatic nerve specimen was perfused by an oxygenated Tyrode solution (mM: NaCl, 145, 0; KCl, 4, 0; MgCl_2_, 1, 0; CaCl_2_, 1, 80; Tris, 5. 0; Glucose, 10. 0; pH 7.3–7.5) containing 10^−3^, 10^−4^, 10^−5^, or 10^−6^ M concentrations of LHT-15-32 at 22–24°C. To create moderate acidosis, the pH of the Tyrode solution was adjusted to 6.5–6.7 by HCl. The specimen was stimulated by linear impulses of increased current strength using gold-plated brass electrodes (ADInstruments, USA) and a BIOPAC MP-160 system. Action potential (AP) amplitude was measured and expressed as % of baseline.

### 2.10. Na^+^ Channel Blocking Activity

We used whole-cell configuration of the patch-clump method to access the ion current through Na^+^ channels of a nondifferentiated neuron. The neurons were dialyzed with an oxygenated solution (mM: CsCl, 120; Tris-OH, 2 with pH 7.3–7.4). Outer membrane surface was perfused by the LHT-15-32 solution (mM: NaCl, 110; MgCl_2_ and Tris-OH, 2 with pH 7.5) [9].

### 2.11. Morphological Evaluation

To evaluate local morphological changes, soft tissues around the impacted molar of rats from intact, control, and main groups, overdosed with thiopental sodium, were examined five days after LA assessment. Microscope slides were made by the standard procedure: tissues were fixated in 10% neutral buffered formalin and embedded in paraffin (Leica LP 1020 Tissue Processor, Germany). Then, sectioning of paraffin blocks was performed (Leica RM 2265, Germany) with following deparaffinization and staining with hematoxylin and eosin. Three-micrometer tissue slides were viewed through a light microscope Leica with a digital camera DMC5400 (Germany).

### 2.12. Statistical Analysis

Statistical processing of data obtained was carried out using SPSS (version 16.0) [10]. Continuous variables were presented as mean (M) value ± square deviation (SD). The normality of distribution was determined by one-way analysis of variance (ANOVA). Newman–Keuls parametric criterion was used for intergroup comparison. LD_50_ indexes were calculated by probit analysis [6].

## 3. Results and Discussion

### 3.1. Results

**Figure 1:**
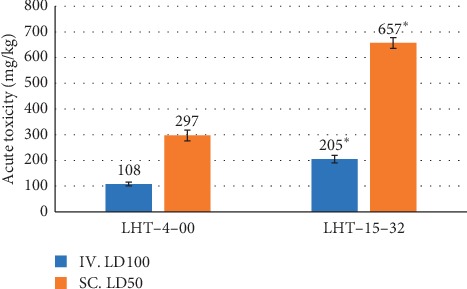
Acute toxicity of LHT-15-32 and its active substance in mice: ^*∗*^*P* < 0.05 when compared with LHT-4-00 (ANOVA).

Acute toxicity of the IV administered formulation for mice was 205 ± 7 mg/kg vs. LD_100_ index of its active substance alone averaged 108 ± 12 mg/kg (*P*=0.003, Figure 1). LD_50_ indexes of both the formulation and its active component LHT-4-00 for the SC route were significantly higher (657 ± 21 mg/kg and 297 ± 20 mg/kg for LHT-15-32 and LHT-4-00, respectively) than for IV route of administration.

**Figure 2:**
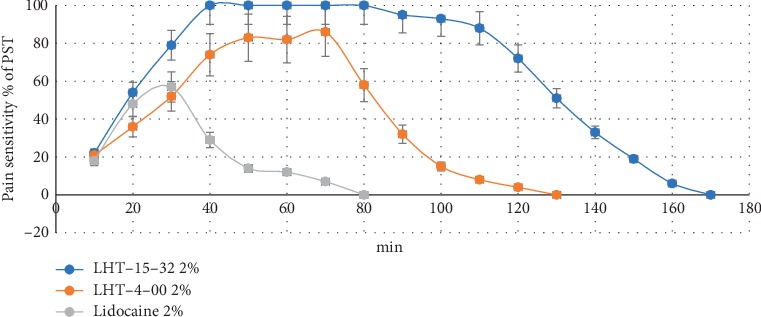
Upper molar pain sensitivity dynamic in rats with periodontitis after 2% LHT-15-32 or referent drug infraorbital injection.

Infraorbital injection of lidocaine led to a moderate dental anaesthesia onset in rats with periodontitis which lasted no long than 17.3 ± 2.3 min (Figure 2). LHT-4-00 deeply anaesthetized the tooth with lower rapidity of anaesthesia embarking. The duration of painkilling averaged 32.4 ± 3.4 min (*P*=0.001 when compared with lidocaine). The formulation induced full anaesthesia onset 32.1 ± 1.7 min after infraorbital injection. Rats of the main group did not react on painful incentives during 72.1 ± 2.6 min of stimulation (*P*=0.001 when compared with both lidocaine and LHT-4-00).

OSA level in the gingival mucosa of rats with periodontitis increased more than thrice when compared with intact animals (Table 1), whereas TAC proportionally decreased. All tested solutions one day after infraorbital administration caused changes in local oxidative stress activity. OSA in the gingival tissue of rats which received lidocaine decreased to 6.4 ± 0.7 × 10^3^ imp/s, LHT-4-00 to 5.3 ± 0.4 × 10^3^ imp/s, and LHT-15-32 to 3.2 ± 0.3 × 10^3^ imp/s (*P*=0.03 when compared with lidocaine). TAC value in the lidocaine group was 1.7 ± 0.4 × 10^3^ imp/s, in the LHT-4-00 group 3.9 ± 0.3 × 10^3^ imp/s, and in the main group 4.7 ± 0.5 × 10^3^ imp/s (*P*=0.005 when compared with lidocaine). Periodontitis was associated with both IL-1*β* and TNF-α elevation along with depression of IL-10 tissue level. Unlike the lidocaine group, in the main group, IL-1*β* and TNF-α tissue levels significantly decreased while IL-10 concentration remained unchanged (Table 1).

**Figure 3:**
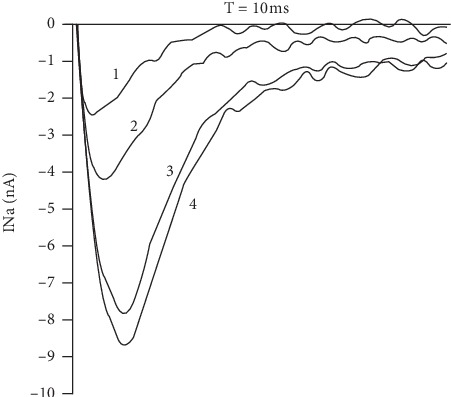
LHT-15-32 inhibits Na^+^ current (INa) through the membrane of isolated *Limnaea stagnalis* neurons perfused by the formulation-containing solution (1, 10^−3^; 2, 10^−4^; 3, 10^−5^; 4, 10^−6^); *n* = 6 in each experiment.

**Figure 4:**
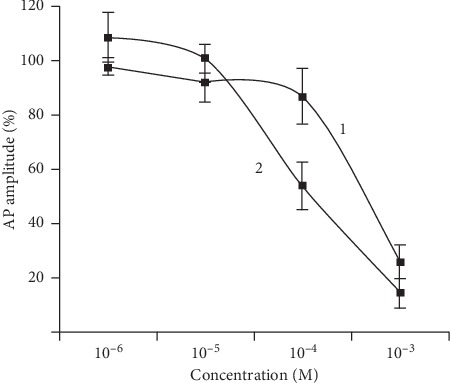
Concentration-related depression of AP amplitude (% of baseline) of *Rana radibunda*'s sciatic nerve perfused by LHT-15-32 under pH 6.5 (1) and 7.4 (2), *n* = 6 in each experiment.

Sciatic nerve specimen AP amplitude replied with decrement on perfusion with the LHT-15-32-containing solution both under physiological and moderate acidosis condition (Figure 3). LHT-15-32 at 10^−6^ to 10^−3^ M concentration applied at the outer side of the neuronal membrane in a dose-dependent manner blocked Na channels of isolated neurons (Figure 4) with calculated EC_50_ 7.78 × 10^−5^ M.

**Figure 5:**
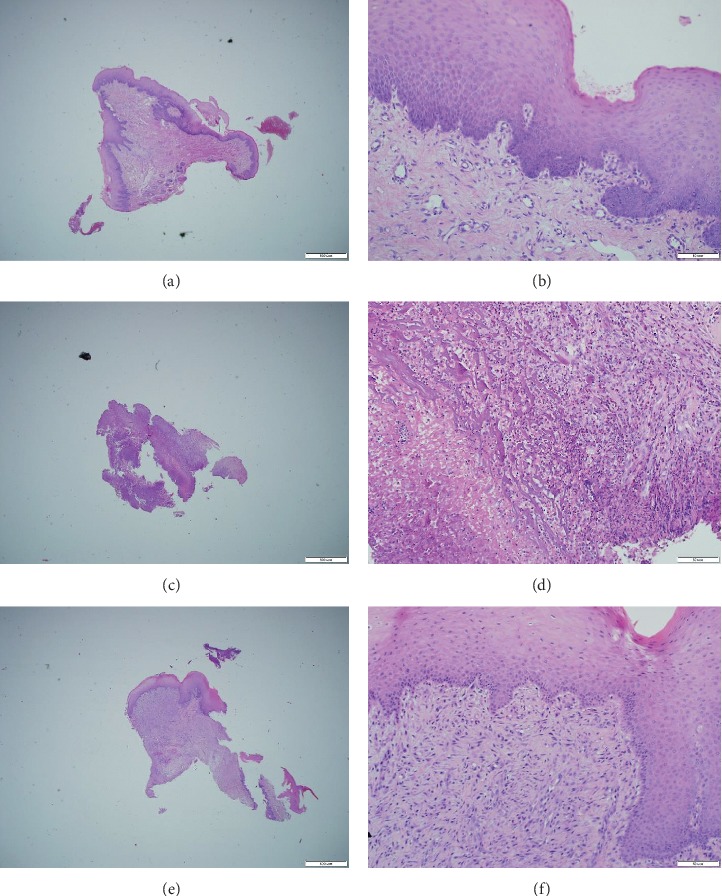
Local morphological changes in soft tissues of intact rats (A and B), animals of the control group (C and D), and rats of the main group (E and F) five days after LA assessment. Hematoxylin and eosin staining method was used. (a) Intact rat, ×5. (b) Intact rat, ×200. (c) Rat of the control group, ×5. (d) Rat of the control group, ×200. (e) Rat of the main group, ×5. (f) Rat of the main group, ×200.

Morphological evaluation of gingival tissues of animals with intact teeth showed no changes: mucous membrane was covered with a normal squamous epithelium. Submucosa was composed of connective fibers, fibroblasts, and capillary vessels with limited number of lymphocytes. Periodontal ligament was preserved (Figures 5(a) and 5(b)). Experimental periodontitis was manifested by neutrophil infiltration and periodontal tissue lysis (Figures 5(c) and 5(d)). Infraorbital administration of LHT-15-32 triggered soft tissue reparation around the impacted upper molar in rats of the main group (Figures 5(e) and 5(f)). We observed that it was covered with squamous epithelium mucous membrane. Submucosa and periodontal ligament contained mature granulation tissue with numerous fibroblastic cells, lymphocytes, macrophages, and capillary type vessels.

### 3.2. Discussion

Many practitioners have been experiencing failure of LA action in anesthetizing teeth of patients with periodontitis. Increasing discomfort during manipulation can even lead to bad clinical outcomes and unsatisfactory results [11]. Several reasons have been proposed to explain the lack of LA effect related to the inflammatory process in dental practice. Among them, (a) effect on the peripheral vascular system; (2) damage to nociceptors due to local oxidative stress activation; and (3) reducing the sensitivity of biological targets of LA has the greatest importance [1]. Novel formulation LHT-15-32 has been developed to address some of them, and therefore enhance efficacy and safety of pain control in dental practice.

The formulation contains N-acetyl-L-glutamic salt of dimethylacetamide (lidocaine) (LHT-4-00), with improved pharmacological properties (such as depth and duration of LA efficacy), as an active component [12]. The study results demonstrate prolonged LA effect of the formulation in animals with periodontitis in comparison not only with lidocaine but with LHT-4-00: LHT-15-32 induces a full anaesthesia onset 32.1 ± 1.7 min after infraorbital injection with no less than 72.1 ± 2.6 min of its duration. As *in vitro* experiments show, LHT-15-32 depresses isolated nerve conductivity at a broad variety of concentrations (10^−6^–10^−3^ M) applied at the outer side of the neuronal membrane blocks Na channels in a dose-dependent manner. Nevertheless, satisfactory pain control in this case cannot be explained only by Na^+^ channel blocking and nerve conductivity depression because both reference drugs act similarly [13].

Plausible explanations may be associated with antioxidant and vasoconstrictive components of the formulation. Tsuchiya et al. have demonstrated direct interrelation between cellular membrane oxidative stress and LA activity [1]. LHT-15-32 more effectively balances OSA and TAC in rats' gingival tissue than its active component as monotherapy. It is noteworthy that reduction of local periodontal and gingival inflammatory process may be directly associated with as LA improvement as a medication safety [14]. Cunha et al. have shown the critical role of IL-1*β* and TNF-α in inflammation-associated hyperalgesia development [13], while Zhang et al. have reported sufficient involvement of IL-10 in alveolar bone restoration in patients with periodontitis [15]. Decreased IL-1*β* and TNF-α level one day after LHT-15-32 injection shows the drug involvement in cytokine regulation, and hence approves anti-inflammatory property of the drug. Acute toxicity data combined with local morphological changes at the site of action may supply the researchers with valuable information about LA drug safety. Acute toxicity of LHT-15-32 was significantly low than that of its active substance LHT-4-00 as via intravascular and subcutaneous route of administration. At the same time, morphological examination of soft tissue around the impacted tooth shows features of triggered repair in the group of animals that underwent LHT-15-32-induced LA five days before.

However, the proposed formulation is not without limitation. Tissue and LA drug solution pH is a critical point and of not less importance for LHT-15-32. Thus, to achieve effective pain control, pH of the formulation should be adjusted *ex tempore*, which is associated with some inconvenience.

On the whole, the proposed formulation may be considered as an effective and safe tool for anesthetizing teeth with inflammatory changes.

## 4. Conclusions

Novel formulation LHT-15-32 is less toxic than its active substance and lidocaine, induces deep and long infraorbital anaesthesia of rats' upper molar with periodontitis, depresses oxidative stress reaction and inflammatory process in gingival tissue, and inhibits isolated nerve conductivity under the condition of moderate acidosis due to Na^+^ current blocking. Therefore, the formulation may be considered as an effective and safe approach to dental anaesthesia in periodontitis.

## Figures and Tables

**Table 1:** Local oxidative stress activity and tissue IL-1*β*, IL-10, and TNF-α concentration in the gingival mucosa of rats with periodontitis one day after LHT-15-32 infraorbital application.

Group of animals	Chemiluminescence, × 10^3^ imp/s		Cytokine level, pcg/g		
	OSA	TAC	IL-1*β*	IL-10	TNF-α
Intact group, *n* = 10	2.8 ± 0.2	3.1 ± 0.3	0.3 ± 0.1	15.8 ± 0.5	12.4 ± 1.8
Control, *n* = 10	8.3 ± 0.4^*∗*^	1.0 ± 0.2^*∗*^	3.2 ± 0.4^*∗*^	3.6 ± 0.3^*∗*^	129.3 ± 4.9^*∗*^
2% lidocaine, *n* = 10	6.4 ± 0.7^*∗*^	1.7 ± 0.4	3.0 ± 0.2^*∗*^	4.7 ± 0.4^*∗*^	110.6 ± 3.5^*∗*^
2% LHT-4-00, *n* = 10	5.4 ± 0.4^*∗*#^	3.9 ± 0.3^†#^	2.7 ± 0.3^*∗*^	3.9 ± 0.3^*∗*^	103.9 ± 4.1^#*∗*^
2% LHT-15-32, *n* = 10	3.2 ± 0.3^†#^	4.7 ± 0.5^†#^	0.9 ± 0.1^*∗*†#^	4.3 ± 0.5^*∗*^	23.7 ± 2.3^†#^^*∗*^

Note: *P* < 0.05; ^*∗*^when compared with the intact group; ^#^when compared with the control; ^†^when compared with the lidocaine group (ANOVA; Newman–Keuls criterion).

## Data Availability

The abstract of invention on the LHT-4-00 substance used to support the findings of this study has been deposited in the Russian Federal Service for Intellectual Property repository (http://www1.fips.ru/ofpstorage/Doc/IZPM/RUNWC1/000/000/002/657/613/ИЗ-02657613-00001/DOCUMENT.PDF).
